# Risk factors for readmission for phototherapy due to jaundice in healthy newborns: a retrospective, observational study

**DOI:** 10.1186/s12887-020-02157-y

**Published:** 2020-05-26

**Authors:** Amit Blumovich, Laurence Mangel, Sivan Yochpaz, Dror Mandel, Ronella Marom

**Affiliations:** Department of Neonatology, Lis Maternity Hospital, Tel Aviv Sourasky Medical Center, affiliated to the Sackler Faculty of Medicine, Tel Aviv University, 6 Weizmann St, 6423906 Tel Aviv, Israel

**Keywords:** Neonatal jaundice, Readmission, Phototherapy, Hospitalization stay

## Abstract

**Background:**

The guidelines of the American Academy of Pediatrics (AAP) for monitoring neonatal jaundice recommend universal postnatal screening for hyperbilirubinemia within 48 h from discharge. We observed that neonate with low-risk jaundice were more likely to be readmitted to hospital for phototherapy compared to neonate with high-risk jaundice. The aim of this study was to identify additional factors that increase the risk for jaundice-related readmission.

**Methods:**

This observational case-control study was performed on 100 consecutive neonates with jaundice who were readmitted to hospital for phototherapy treatment and were compared to 100 neonates with jaundice during hospitalization who were not readmitted after discharge. The data retrieved from the medical records of all participants included maternal characteristics, delivery type and noteworthy events, gestational age at delivery, birth weight and weight loss, neonate physical findings, Apgar scores, laboratory findings, length of hospital stay, and administration of phototherapy during hospitalization. The length of time since discharge and readmission for jaundice was also recorded.

**Results:**

The risk of readmission decreased by 48% [odds ratio (OR) =0.52; 95% confidence interval (CI) 0.341–0.801] with every day added to the original hospitalization stay, and by 71% (OR = 0.29; 95% CI 0.091–0.891) if phototherapy had been administered during postnatal hospitalization. In contrast, the risk increased by 28% (OR = 1.28; 95% CI 1.164–1.398) with every elevation by 1% in hematocrit, and by 2.78 time (95% CI 1.213–6.345; *p* = 0.0156) when the delta in infant weight was > 5% (the difference between birth weight and weight at discharge during the postnatal hospitalization).

**Conclusions:**

The risk factors for readmission, such as substantial weight loss (> 5% difference between birth and discharge) and elevated hematocrit should be taken into account in the decision to discharge neonate with low-risk jaundice. The AAP guidelines for decreasing readmission rates of neonatal jaundice by postnatal screening for hyperbilirubinemia alone may be more appropriate for neonate with high-risk jaundice.

## Background

Postnatal hospital stay in many developed countries has been decreasing over the past few decades [1, 2]. In Israel, the national health insurance covers a hospital stay of 48 h after an uneventful vaginal birth and a stay of 3 days after an uncomplicated caesarean delivery as the accepted length of stay (LOS) for mothers and infants. A similar trend was observed in a Canadian study that evaluated close to 2 million live births and found that approximately 47% of mother-infant dyads were hospitalized for 1 day following a vaginal birth and that approximately 49% were hospitalized for 3 days following a caesarean delivery [3]. While there are many benefits to early discharge, including cost reduction, higher availability of hospital beds for other mothers [1], and reduced risk for nosocomial infections to mothers and infants, there are also specific risks associated with premature discharge, even of healthy infants, with hyperbilirubinemia being chief among them [4]. Physiological jaundice typically appears in over 80% of newborn babies. It develops between the second and fourth day of life and peaks between the fourth and fifth day [5], thus, under current practices, it is most likely to peak after the neonate has left the hospital.

A recent study conducted in the UK analyzed over 4 million live births and reported a readmission rate for neonatal jaundice of 5.2% [2]. A Canadian study observed that 4.2% of infants following a vaginal birth and 2.2% following a caesarean delivery were readmitted for neonatal jaundice [3]. The findings of analyses that compared length of postnatal stay and readmission for neonatal jaundice have been inconclusive, however, the correlation appeared to be stronger for term infants than for late preterm infants [2–4]. Guidelines issued in 2004 by the American Academy of Pediatrics (AAP) [6] and updated in 2009 [7] on the follow-up and management of infants post-discharge recommended a visit at a healthcare provider within 48 h from discharge. The Israeli guidelines on hyperbilirubinemia management also include a recommendation for all infants to be seen by a healthcare professional within 2–3 days post-discharge, irrespective of total serum bilirubin levels at discharge and the presence or absence of risk factors for developing severe hyperbilirubinemia [8]. Accordingly, several reports suggested that implementation of universal postnatal screening for hyperbilirubinemia within 48 h from discharge might decrease hospital readmission rates due to neonatal jaundice [9, 10]. However, it was our impression that there were additional factors that increased the risk for jaundice-related readmission. The aim of the present study was to identify those risk factors.

## Methods

### Setting and study population

This work is a retrospective observational case-control study. We reviewed the medical records of all term and late preterm neonates admitted to the Lis-Maternity hospital at the Tel Aviv Medical center between January 2015 and March 2016. Our institute hosts 12,000 births per year. We used the diagnosis key word “readmission” to recruit the study cases defined as neonates with jaundice in their first hospitalization and readmitted for phototherapy. Only 100 consecutive cases of neonates readmitted to the newborn nursery for phototherapy treatment were retrieved (study group). To these study cases, 100 neonates who had neonatal jaundice during hospitalization but were not readmitted to the hospital for phototherapy (control group) were matched. The subjects in the control group were matched on the basis of gestational age (GA) and birth date (±7 days) to the subjects of the study group. We included neonates presenting solely with neonatal jaundice and no additional morbidities.

### Data collection and handling

The collected data included maternal and infant demographics, clinical and laboratory information, and details on the course of jaundice. The study was approved by our local Institutional Review Board which waived informed consent.

### Statistical methods

Risk factors for readmission were compared by means of univariate analyses between newborns that were readmitted and those who were not. A 2-sample Student’s t-test or Wilcoxon 2-sample test was applied for continuous variables (depending upon the normality of the distribution), and the χ^2^ test for categorical variables. A multivariate logistic regression was applied to identify the significant independent predictors of readmission by considering predictor variables whose associated *p* value in the univariate analysis was < 0.05 or if the variable was thought to be clinically relevant. A backward stepwise selection procedure was used to establish the final multivariate model. A 20% significance level of the χ^2^ score was selected for entering an effect into the model, and a 10% significance level of the Wald χ^2^ for an effect to stay in the model. The statistical analysis was performed using the SAS software version 9.4 for Windows.

## Results

The study included data on 200 infants, of whom 100 were in the readmission group (study group) and 100 were in the no-readmission group (control group). The average maternal age in both groups was approximately 33 years, the median maternal parity was 2.0 in both groups (Q1, Q3; 1.0, 3.0) (Table 1), and the median GA was 38 weeks (Q1, Q3; 37.0, 39.0) (Table 2).

**Table 1:** Delivery and Maternal Characteristics in the Study and Control Groups

Variables	No-Readmission Control Group (*N* = 100)		Readmission Study Group (*N* = 100)		*p* Value
Maternal age, mean (SD)	33.1	(4.9)	33.4	(4.3)	0.66
Parity, median (Q1, Q3)	2.0	(1.0, 3.0)	2.0	(1.0, 3.0)	0.53
Gravidity, median (Q1, Q3)	1.5	(1.0, 2.0)	2.0	(1.0, 3.0)	0.24
Gestational diabetes mellitus	17	(17%)	11	(11%)	0.22
Meconium-stained amniotic fluid	12	(12%)	10	(10%)	0.65
Group B streptococcus	5	(5%)	8	(8%)	0.39
Hypothyroidism	5	(5%)	7	(7%)	0.55
Other^a^,	11	(11%)	6	(6%)	0.21
Multiple gestations	2	(2%)	1	(1%)	0.56
Mode of delivery					0.003
Vaginal	70	(70%)	83	(83%)	NS
Vacuum	12	(12%)	14	(14%)	NS
Caesarean	18	(18%)	3	(3%)	< 0.001
Rupture of membranes	19	(19%)	15	(15%)	0.45
Positive Coombs	23	(23%)	1	(1%)	< 0.001

^a^Other - RH factor, in-vitro fertilization, use of selective serotonin re-uptake inhibitors or pre-eclampsia, *NS* non-significant, Data are expressed as n (%),

**Table 2:** Infant- and Jaundice- Related Characteristics in the Study and Control Groups

Variable	No Readmission Control Group (*N* = 100)		Readmission Study Group (*N* = 100)		*p* Value
Female, n	49	(49%)	41	(41%)	0.26
Birth weight, gr, mean (SD)	3201.0	(434.7)	3163.3	(416.7)	0.53
Gestational age (weeks), median (Q1, Q3)	38.0	(37.0, 39.0)	38.0	(37.0, 39.0)	1.00
Late preterm neonate (< 37), n	8	(8%)	8	(8%)	1.00
Size for gestational age, n (%)					0.41
Appropriate	81	(81%)	86	(86%)	
Large	15	(15%)	9	(9%)	
Small	4	(4%)	5	(5%)	
G6PD deficiency, n (%)	14	(14%)	14	(14%)	1.00
Apgar 1 (range 5–7), n	7	(7%)	2	(2%)	0.09
Apgar 5 (range 5–7), n	1	(1%)	0	(0%)	0.32
Feeding, n					0.11
Breastfeeding	49	(49%)	51	(51%)	0.78
Mixed	39	(39%)	45	(45%)	NS
Formula	12	(12%)	4	(4%)	0.037
Postnatal LOS, median (Q1, Q3)	4.0	(3.0, 5.0)	2.0	(2.0, 3.0)	< 0.001
Weight loss > 5% n (%)	42	(42%)	57	(57%)	0.034
Laboratory findings during first hospitalization					
Highest value of total bilirubin, umol/L, mean (SD)	13.4	(2.3)	11.9	(2.9)	< 0.001
Hemoglobin, g/dl, median (Q1, Q3)	17.6	(16.0, 19.2)	19.6	(18.8, 21.4)	< 0.001
Hematocrit (%), median (Q1, Q3)	53.0	(48.0, 58.0)	59.0	(56.0, 63.0)	< 0.001
Reticulocyte > 5%, n	40	(40%)	9	(9%)	< 0.001
Highest value of total bilirubin during second hospitalization	.		17.9	(1.8)	.
Visits to jaundice clinic, median (Q1, Q3)	1.0	(0.0, 2.0)	3.0	(2.0, 4.0)	< 0.001
Phototherapy during first hospitalization, n	63	(63%)	10	(10%)	< 0.001

*NS* non-significant, *LOS* length of stay

### Delivery and maternal factors (univariate analyses)

Table 1 lists the selected factors related to maternal demographics and clinical characteristics that were assessed. There were significant differences between the study and control groups in prevalence of caesarean delivery (3 and 18%, respectively; *p* <  0.01) and in the prevalence of a positive Coombs test (1 and 23%, respectively; *p* <  0.01) (Table 1).

### Infant and jaundice-related factors (univariate analyses)

The results of the evaluations of selected clinical characteristics of the infants as well as those specific to jaundice and its management are presented in Table 2. There were significant differences between the groups in formula feeding (4 and 12% in the study and control groups, respectively; *p* = 0.037) and postnatal hospital length of stay (LOS) (medians 2 days and 4 days, respectively; *p* <  0.001). In addition, the control group was significantly different from the study group in terms of infant weight loss (i.e., the difference between birth weight and weight at postnatal discharge) above 5% (57% in the study group and 42% in the control group; *p* = 0.034). Laboratory findings (total bilirubin, hemoglobin, hematocrit, and reticulocytes), number of visits at the outpatient jaundice clinic (a median of 1 in the study group and 3 in the control group; *p* <  0.001), and phototherapy during the first hospitalization (10% versus 63%, respectively; *p* <  0.001) were also significantly different between the groups.

### Multivariate analyses

The multivariate analyses revealed that the risk of readmission decreased by 48% with every day added to the postnatal hospital LOS (OR = 0.52; 95% CI 0.341–0.801; *p* = 0.0029), and by 71% if phototherapy was provided during that hospital stay (OR = 0.29; 95% CI 0.091–0.891; *p* = 0.0308). In contrast, the risk increased by 28% (OR = 1.28; 95% CI 1.164–1.398; *p* <  0.0001) with every 1% elevation in hematocrit, and by 2.78 time (95% CI 1.213–6.345; *p* = 0.0156) with an infant weight loss greater than 5% (Table 3).

**Table 3:** Multivariate Analysis

Parameter	Category	Odds ratio	95% Wald Lower CI	95% Wald Upper CI	*p*-value
Hospital LOS after birth (days)		0.52	0.341	0.801	0.0029
Hematocrit		1.28	1.164	1.398	< 0.0001
Weight loss > 5%	Yes vs. No	2.78	1.213	6.345	0.0156
Phototherapy during first hospitalization	Yes vs. No	0.29	0.091	0.891	0.0308

*LOS* length of stay

## Discussion

In this study, we analyzed various potential risk factors for hospital readmission of newborns for phototherapy due to jaundice following discharge. The results of the analyses revealed that the length of postnatal hospital stay and the administration of phototherapy were significantly associated with a lower risk for readmission. Our medical center adheres to the Israeli guidelines for the management of neonatal jaundice [8, 11]; which are based on the AAP guidelines [6]. Implementing guidelines for monitoring hyperbilirubinemia and universal screening for bilirubin have proven effective in reducing the overall rate of readmission for treating jaundice in the high-risk group [4], such as, preterm infants, neonates with early jaundice during the first 24 h of life, neonates with ABO incompatibility and positive coombs test or other hemolytic disease (eg, G6PD deficiency) [9].Hence, the neonates in the No-Readmission group had longer hospitalization stay due to ABO incompatibility or preterm jaundice that needed phototherapy treatment and which finally was associated with a significantly reduced risk of readmission.

Our data suggest that the neonates in the Readmission group have been assessed as having none of the major risk factors for developing hyperbilirubinemia and as being in the low-risk zone according to the AAP guidelines and therefore discharged early [7]. In fact, these newborns were not at such a low-risk and experienced a post-discharge elevation of bilirubin leading to readmission for phototherapy treatment.

Several studies reported a correlation between the status of a newborn as a “late preterm” and increased risk for readmission [2, 7, 12]. There was no comparable correlation in our study population, most probably due to the extended hospitalization stay of late preterm newborns as in the high risk group. The same discrepancy between the findings of others and our current ones was noted with respect to levels of bilirubin at discharge [13]. It is possible that an intensive post-discharge management contends with that risk and offsets the need for readmission. Interestingly, we found an increased risk of readmission associated with a shorter LOS. This is in line with the study of Ruth et al. work who found that a birth stay of ≤2 calendar days increased the risk of readmission and the magnitude of that risk remained unaffected by infant GA [14]. Similarly, Jones et al. found that the vast majority of infants (94%) admitted for physiological jaundice had a hospital duration of ≤3 days [15].

Furthermore, we showed that an elevated hematocrit at discharge was also associated with a higher risk of readmission, possibly reflecting polycythemia among the readmitted infants. Even when the hematocrit is within a physiologic range such as in Cernadas et al. study [16]. Indeed, Mimouni et al. reported that polycythemia was associated with hyperbilirubinemia via the breakdown of the increased mass of red blood cells [17]. The elevated hematocrit observed in the current study group may partly be a consequence of a delay in umbilical cord clamping. Such an association between late cord clamping and jaundice [14] through elevated hematocrit was demonstrated by McDonald et al. [18].

In line with the known association between weight loss and hyperbilirubinemia [6], substantial weight loss (the difference between birth weight and discharge weight) was a significant risk factor for readmission in our population. This finding is consistent with the study by Campbell Wagemann et al. which showed that the main risk factor for readmission due to severe hyperbilirubinemia was excessive weight loss in newborn between 4 and 7 days after birth [19]. However, contrary to our expectations, there was no significant difference in post-delivery weight loss between breastfed babies and formula-fed babies. Yet, a weight loss above 5% remained as an independent risk for readmission (logistic regression-Table 3).

Newborns with jaundice should have bilirubin levels closely monitored before and after discharge from the hospital to prevent potentially serious complications of hyperbilirubinemia. The clinical practice guideline published by the American Academy of Pediatrics (AAP) in 2004 recommended that all neonates born at ≥35 weeks of gestation be assessed before discharge for the risk of severe hyperbilirubinemia by using clinical risk factors and/or bilirubin measurements. Weight loss nor hematocrit level are among the criteria included in the assessment before discharge for the risk of severe hyperbilirubinemia in the AAP guidelines. We suggest that Both of these criteria should be considered in deciding whether to release home in order to prevent readmission.

This study has some limitations that bear mention. This is a retrospective study on a small cohort. In addition, since we only evaluated infants diagnosed with jaundice prior to their postnatal hospital discharge and not the entire infant population before discharge during the study period, our results cannot be generalized. Further research is warranted to support these preliminary findings.

## Conclusion

In the present study, we identified the haematocrit level, an infant weight loss > 5%, and a shorter LOS as being additional risk factors for increased risk of readmission of low-risk neonates presenting with physiological jaundice. We suggest that while the AAP guidelines are appropriate for the management of high-risk neonates presenting with jaundice, they are less suitable to low-risk neonates presenting with physiological jaundice. We conclude that the criteria for hospital discharge of the latter neonates need to be more stringent.

## Data Availability

The datasets used and/or analyzed during the current study are available from the corresponding author on reasonable request.

## Abbreviations

LOS: Length of stay
AAP: American Academy of Pediatrics
